# On Life, from a Physical Basis

**Published:** 1870-01

**Authors:** Z. C. McElroy

**Affiliations:** Zanesville, Ohio, President Muskingum County Medical Society


ARTICLE II. — *On Life, from a Physical Basis; and some Qualities or Phenomena, which are thought to be Peculiar to Life, and Irreconcilable with Physics.* By Z. C. MCELROY, M.D., Zanesville, Ohio, President Muskingum County Medical Society.

THE qualities possessed and phenomena developed by human beings and other organized bodies, and the qualities and phenomena of inorganic matter on their surfaces, present few points of resemblance. Observers, from the remotest antiquity, have all, or mainly all, united in attributing certain phenomena to an unknown force, or something, which they designated as "vitality," "vital force," or "vital principle," or "life." Its existence was inferred from the phenomena of life, and the qualities and phenomena possessed by living beings were admitted to demonstrate its existence.

Doubters may, perhaps, have existed, from time to time, of this philosophy; but, if so, they failed to make any impress on current science or thought, until a very recent period. But they do exist now, and though their numbers may be few, they will not likely ever be any less.

In this iconoclastic movement, the Muskingum County (Ohio) Medical Society is entitled to whatever merit may hereafter attach to the initiative, or the dishonor attached to failure, in endeavoring to bring the phenomena of organic life, in health and disease, squarely within the pale of physical or exact science, and the dethronement of the imaginary idol of "vitality," as a superterrestrial force, which has so long swayed the minds of scientific investigators of the material and forces of organic life.

But its membership is, by no means, a unity in the matter; while all admit, perhaps, that some of the phenomena of organic life may be due to the operation of the ordinary physical forces of nature, there are others who think that living beings possess some qualities, which, in themselves, are peculiar, and irreconcilable with any ideal of life from an exclusively physical basis. Thus, at a recent meeting, one of its members read an elaborate, carefully-studied, able and dignified paper, opposed to a physical basis of life, and summing up the following qualities as points for which physical science offered no explanations:*

"But, allow me to mention some qualities which are peculiar to life, viz.:

1st. Activity: contra distinguished from simple motion, and this presenting often as opposed to the "physical forces."

2nd. Individuality; which causes even like particles of matter to present different features.

3rd. Reproduction; or perpetuation of individuality.

4th. Immutability; or the maintenance of "life," notwith-

* Copy furnished me by the author.

standing all the molecular changes which take place in an organic body.

5th. Sensation; or the appreciation of bodily states and wants.

6th. The quality of Perception.

7th. The quality of Instinct; and, in man,

8th. The faculty of Reason;

9th. The faculty of Consciousness;

10th. The Will, and mental and moral phenomena in general."

These are strong points, well and wisely chosen, and if they cannot be reduced to simple physical elements, and shown to have their manifestations due to the operations of ordinary chemical and physical law, the attempt to construct a physical basis of life has been undertaken without sufficient knowledge, and must be considered a failure. It will, therefore, be needful to analyze each of these ten qualities separately, and ascertain whether they can be reduced to simpler elements than appear on their surfaces.

1st. Activity, and "as contradistinguished from simple motion." Activity, if it has any meaning at all, is rather a quality of motion than anything distinct from motion. It is, in fact, motion — nothing more, nothing less — if it represents any thing else than a quality of motion; and, in organic life, is certainly due to correlations of the ordinary physical forces of light, heat, electricity, etc., etc. It seems to me quite unscientific to invent causes, when those already in actual existence are at hand to account for any or all of the qualities or phenomena of "life." The simplest element therefore of activity is motion.

3rd. Individuality. That is, separate and distinct existence. The wheaten loaf is certainly the formless protoplasm of all animal tissues; that is, man and beast, bird and worm, fish or insect — the most diversified distinct existences — may eat from the same wheaten loaf, and at will make their tissues; that is, their separate existences. Hence, their individuality must consist in something else than matter, for the same first matter of life enters into them all; and the simplest element, therefore, of individuality must be in form, or forms. Thus, the difference between bone and brain matter is certainly very striking, yet that difference is due to form, and not to the material of which each is composed; for, in different proportions, probably, their ultimate elements are the same. Nor is the resemblance between the human infant, deriving its whole sustenance from its mother's milk, and the mother's milk, any nearer; yet the tissues of the infant have certainly been constructed from its mother's milk; and the difference between them is principally in their forms. Again, the material of the pen which traces the manuscript from which this article is printed, existed, possibly, at one time, in combination with quartz rock in California. It was gold then, and is gold still. After passing through many metamorphoses, it has become a pen, and the feature which distinguishes it from the gold from which it was made, is its form. The simplest element, therefore, of individuality is form.

4th. Reproduction. In organic life, no one expects, because such things have never happened to any one's knowledge, that in the act of reproduction, an elephant should give birth to a chicken, for example, or to perpetuate any other than its own general forms. Hence, reproduction, stated in its simplest terms, consists in form and motion.

5th. Immutability. Maintenance of form with incessant, or, perhaps, frequent molecular changes. The simplest statement of immutability, as applied to the maintenance of life or form, is form; and is due to the operations of what the writer has designated as the "form force" of organic life.* But the records of morbid anatomy unhappily show that organic forms fall short of immutability; for if the forms of organic life were really and truly immutable (in the ordinary meaning of the word); that is, always maintaining "life" or "forms," organized bodies would be immutable, and, in addition, possess another quality, to-wit: immortality. These would be, because there could be neither disease, old age, or death; for these are due, taking the testimony of pathology and morbid anatomy to (in their phraseology) changes of structure, which is, again, simply changes of form. For if the forms of organic life were always maintained intact, the vocation of the physician would as certainly cease as the vocation of Demetrius† ceased with the worship of Diana. The simplest element of immutability, as here used, is form.

* "Western Journal of Medicine," September, 1869.

† Acts of the Apostles, xix. 27.

5th. Sensation. That is, sight, hearing, touch, taste, smell, hunger, thirst, ease, pain, etc., etc. For the appreciation of these, or any other bodily states or wants, taking the testimony, again, of anatomy and pathology, a nerve or brain-like mass or patch is necessary, as well as nerve-chords and motion, in their molecular structure or particles. It, therefore, only requires the bare statement, to prove that these states and wants are all reducible to form and motion; for a change of form in the eye, for instance, if to any considerable extent, involves the loss of vision. And this is equally true of all the other organs of sensation. The simplest elements of sensation are, therefore, form and motion.

6th. Perception. That is, knowledge through the medium, or instrumentality of bodily organs. Bodily organs must have form, and motion must occur in their molecular structure, or perception cannot take place. Perception, then, is reducible to form and motion; a cessation of motion is death, and surely dead organic bodies do not perceive.

7th. Instinct. That is, a certain power, independent of all instruction or experience, by which animals are directed to do, spontaneously, without having any end in view, whatever is necessary for the preservation of the individual, or the continuation of its kind. Instinct depends, as demonstrated by both anatomy and pathology, on patches or masses of brain-like matter, and is to be regarded, if regarded on its facts, as any other organic function. Living things are said to be born with instinct; which is true — just as true as that they are born with vision, hearing or digestive apparatus. No human infant has to be taught to see, or hear, or digest proper food. These are the conditions of its life. No living being exhibits what is called instinct, except it has a mass or patch of brain-like matter, which evolves what is called instinct, just as the liver evolves bile; and no one, to my knowledge, has ever thought it necessary to open schools for the purpose of teaching livers to secrete* bile. Instinct, then, is the evolution of masses or patches of brain-like matter, which depend, for the performance of their functions, on form and motion, just as the eye or ear, or liver, or kidneys, or lungs.

* The word "*secrete*" is used, not because it is believed to be properly applied to any organic function. Indeed, it is a most mischievous word in physiology, for the reason that the mental impression produced by the word does not correspond with the facts of life.

Instinct differs from intellection, in that intellection depends, so to speak, on added brain matter, or by culture or education giving the forms to brain-matter from which intellection springs. Hence the necessity of teaching intellection to the young, while brain matter can be given the requisite form, or added; for if delayed till after adult life, these forms are produced with much greater difficulty, as all experience proves, and not unfrequently it is impossible. The proverb that "instruction is for the young," has its foundation in physiological truth. Possibly this fact may account for the difficulty my friend finds in understanding life from a physical basis, instead of a metaphysical, as we were both taught when medical pupils, and probably before.

Instinct, then, is clearly reducible to form and motion.

8th. Reason. That is, to reduce inferences justly from premises. To reason justly, is to infer from propositions which are known, admitted, or evident, the conclusions which are natural, or which necessarily result from them.

Reason is one of the faculties of the intellect, and as reason depends on brain matter, having a definite and peculiar form, and can only be manifested by motion in its molecular structure, this, like all the preceding, is reducible to the two simple physical elements of form and motion.

9th. Consciousness. That is, the knowledge of sensation and mental operations, or what passes in one's own mind. This, like the last, depends on brain matter; that is, form, manifested, like reason, by motion in its molecular structure. It is, therefore, also, merged into the two simple physical elements of form and motion.

10th. The Will, and mental and moral phenomena in general. That is, the faculty of mind by which we determine to do or forbear an action. The will, like consciousness, depends on brain matter, having a definite and peculiar form, and can only be brought within our comprehension by motion in its molecular structure or particles: is one of the intellectual faculties, and as intellect depends on added brain matter, or by culture and education giving the requisite form to brain matter, this, too, like all the preceding, is reducible to the two physical elements of form and motion.

It is seen, therefore, that all the phenomena of organic life, like all the phenomena observed in inorganic matter, are reducible to the two simple physical conditions of form and motion. And however else they may be viewed, metaphysically, must, nevertheless, be studied on their facts alone, by the physical philosopher. The physical conditions for their manifestation are form and motion, and these properties are all that concerns the pathologist and therapeutist. By remedial agents, our power over forms in organic life is *nil;* but over motion, considerable. The management of lost types or forms falls within the province of the surgeon, whose sole power lies in their destruction, when their destruction does not involve life. The surgeon cannot, for example, remove, that is, destroy, an enlarged heart; that is, a heart that has lost its normal form, with any expectation that the patient would survive, even when closing the necessary wounds in the integuments, no matter how expeditious he went about it. But the physician may, by many agents and expedients, prolong life, by complying with the inexorable laws governing lost forms, and, consequently, imperfectly performed function.

The structural unit of animal organic life, is a sac or cell, containing a fluid and a granule. Or the granule or crystal, that is form, may be so regarded. It is a further fact, that the germ is also a sac or cell, containing a fluid and a granule. It may be regarded, therefore, as tolerably certain that the form force is, in some way, identified with the granule or crystal. It may be further regarded as tolerably certain that the material of the germs, for the most diversified forms of life, have, to a great extent, an identity; *i. e.*, that they are composed of the same ordinary inorganic matter, and the physical forces are indispensable for their evolution. A human being is thus complex: 1st. Inorganic matter; 2nd. Physical forces; 3rd. Form force; 4th. Spirit or life.

It is quite probable, and indeed is the present tendency of science, to refer all organic forms to some mode or correlation of the physical forces, operating as one great law or force. The human or living being would then be a triple compound of matter, physical force, and spirit.

But the simplest elements of all animal life, or, for that matter, all organic life, are form and motion; and these forms and motions can only be made manifest to our senses through material common, in all probability, to the universe; for recent scientific investigations have demonstrated the identity of some of the solar and planetary with the inorganic matter of the earth. And it is quite certain that the source of all power is the sun. So that all that is super-terrestrial in the human body is spirit, or, if it is preferred, say "life." But all that we can influence by therapeutic, remedial, or hygienic agencies, are its strictly terrestrial portions. The only way, therefore, in which it can be intelligently studied, for therapeutic and pathological purposes, is from a physical basis; and for the additional reason that when so studied, the facts of health and disease are correctly represented in the mental impressions so obtained.

Diverse, complicated and mysterious as appear the ten specifications of "qualities" peculiar to "life," enumerated by my friend, the analysis of the physical philosopher reduces them all to the same simple, understandable, physical phenomena of form and motion.

Each of the ten several qualities unquestionably depend, for their manifestation, on matter with certain forms, or, in other technology, certainly less definite, structure and motion; and motion as a correlation of the ordinary physical forces of inorganic nature.

Even while writing this short memoir, the *London Lancet* reaches me, with an article on "Granular Degeneration of Nerve Cells in Insanity," by the superintendent of a royal lunatic infirmary in Great Britain; and his facts are not guessed at, but obtained among the dismal yet instructive revelations of the dead-house. He says:

1st. He finds the "grey matter degenerated into fat"—that is, loss of form—"not only where acute mania was present, but in chronic general paralysis." "2nd. In all cases of long standing, the grey matter presents a granular appearance"—loss of form again, and with loss of form, loss of function forever. With the loss of form, several of the ten "qualities" "peculiar to life," so far as these insane patients were concerned, were stricken from the list. 3rd. "That similar granular"—that is, the blood plasma failed to reach any higher organization than what is called tubercular matter—"degenerations are found in and around all parts of the brain and spinal marrow."

These examples serve to show that each and all of these ten separate and apparently distinct "qualities," or phenomena peculiar to "life," are due to the operations of the ordinary physical forces, on the forms of organized inorganic matter; and whoever desires to reduce their phenomena to the simplest possible conceptions will, probably, like myself, find that it is in form and motion.

And furthermore, these two simple physical elements of form and motion embody the central ideals or units of organic life from a physical basis, and explain, in an understandable and intelligent manner, the *modus operandi* of all therapeutic, remedial and hygienic measures whatever. As motion is the central ideal unit of therapeutics, so, loss of form is the central ideal or unit of pathology; or, restricted to its narrowest possible limits, morbid anatomy. All the records of morbid anatomy can be merged into the central ideal of lost forms, and the only difference between the various physical appearances of lost forms, is merged into motion.

Thus, the calx substitutions for tissues of normal form so frequently found in the aged, differ from what is called fungus or cancer, found at all stages of life, solely in their motion. In the calx motion is reduced to its minimum; while, in the so-called cancer or fungus, it is at its maximum, and that maximum much greater than in tissues of normal form. And as motion is the simple unit of force, the active cancer, or fungus, destroys life with much greater certainty and rapidity than lost forms with intermediate grades of motion, as fat, fibrin, etc.

The classification of therapeutic agents,* submitted to you three years since, is thus demonstrated to be critically correct and scientifically accurate. Experience may render some subdivisions expedient, based on their influence on motion, but promoting and retarding constructive and destructive metamorphosis; or otherwise, nutrition and oxidation of the tissues (both being merged into motion), are the simple ultimate physical elements of all therapeutic, remedial and hygienic measures; or, in a wider sense, form and motion are the ultimate, simple physical elements of the forces and phenomena of organic life, thermal, sensory, emotional, chemical and intellectual.

* See "Chicago Medical Journal," March 1, 1869, p. 157.

Over the other ultimate simple physical element of organic life, to wit: forms, neither therapeutic, remedial, or hygienic agencies or measures, exercise other ultimate influence than destruction; which is, again, simply motion. All operative surgery is merged into the ultimate simple physical element of motion, in the destruction of lost forms.

In general pathology, the long list of the so-called diseases are merged into the same simple physical units of lost form and motion. All the so-called diseases, remedial only by surgical proceedings, are merged into the unit of lost forms. All the so-called exanthemata, as small-pox, scarlet fever, measles, etc., as well as the virus of serpents, insects and other animals; cutaneous diseases, so-called, syphilis, etc., are merged into the unit of form changes or forces, and, ultimately, into the simple physical unit, motion; and are brought within the domain of general pathology, in virtue of their power, as form forces, or modes of form force, to substitute their own, or some modification between their own, and the normal forms of human tissues. The whole catalogue of the so-called febrile diseases are merged into the modifications of the molecular arrangements of the normal form and motion of the tissues.

If there is a solitary fact in organic life, physiological, pathological, or therapeutical, which cannot be merged into these simple units of form and motion, it has escaped my scrutiny. As a simple, earnest seeker after truth, no fact, or facts, have been avoided, but each has been squarely faced, and none have been found irreconcilable with life from a physical basis exclusively.

Two cases coming under my professional notice, to-day, of very opposite characters, may be cited in illustration (29th December, 1869):

In consultation. Sarah, aged 8, has been sick, or noticed to be unwell for four days past. 9 o'clock, A.M., temperature 108; pulse 250; respirations 60; pupil dilated; eye globes deeply injected, and lids cement together, in a little while, with matter; tongue purplish red; throat not seen; is very impatient if disturbed; swallows as well as any human being can in her condition; skin covered with minute red dots, in places so closely packed together as to present a continuous scarlet surface. The diagnosis was scarlet fever; and the prognosis unfavorable. Therapeutical indications, tonics, stimulants, and anodyne, to restrain excessive restlessness. Such is a statement of the case in the methods now in use by the profession. Can the phenomena of this case be reduced to simple understandable physical elements? It seems to me that each and every symptom can be merged into nothing else than motion. What is the significance of the temperature, circulation and respiration but motion? And motion at a velocity altogether incompatible with life for many hours, alone. Why this motion? Existing and accepted philosophy declares it to be due to the presence of the poison or virus of scarlet fever in the system. And how does the virus or poison of scarlet fever produce this excessive motion? In no other possible way than by changing forms; for, if the forms of the tissues were undergoing no changes, no such excessive motion could be developed. Then in reference to therapeutics, the word tonic is certainly misleading in producing mental conceptions corresponding with the facts and requirements of the case. It comes from tone, to strain, or stretch, in reference to strings, or wires, whose vibrations produce tones in musical instruments. If by any fanciful use of the imagination the phenomena of this child's case can be compared to the chords of a musical instrument, they will be found, indeed, sadly out of tune; but will straining or stretching help to restore them? Rather is not the simple scientific indication to retard motion? Again, the term stimulants comes from the word stimulate, literally, to goad, or urge on. In medicine, it is true, it is understood in the sense of increasing, temporarily, vital energy or force. But what, it may be asked, is there vital in phenomena of motion presented by this child? Will she be profited, or her chances of life increased by additional energy — that is motion? Surely, such a conception would be most absurd. The facts are that motion, physical motion, is already in excess, and the sole scientific indication is to "retard destructive metamorphosis" of tissue. Once more, in the technology in use by the profession, she would be designated as in a state of great "exhaustion." Exhaustion literally means being deprived of strength, or spirits. Does it indicate exhaustion, that is, emptied, to have a temperature ten degrees above natural? Or for the heart to contract 250 times per minute? Rather are not the phenomena due to terrible energy? Motion incompatible with the continuance of forms, owing to its velocity above the normal standard of human beings? At 6 P.M., nine hours after, the spirit of the little sufferer passed to its celestial abode; and the organized materials of her body given up to other physical motion, in putrefactive decomposition.

T. B. A.; aged 10. Well cared for; goes to school, and stands at the head of her classes nearly all the time; studies hard; eats but little, except cakes, candy, taffy, pies, etc. Has headache; temperature 98½; pulse small in volume, and quicker than natural; skin pale, tongue red, and covered in middle with dark-brown fur; is, however, cheerful and pleasant; bowels sluggish; nothing could be ascertained in regard to renal discharges. The diagnosis of the parents was that she was bilious, and had the neuralgia. Mine was somewhat different. All the symptoms were merged into the unit of motion, which was certainly too rapid in her cerebral masses, as evidenced by the pain above the eyes, and the circulation. The pallor of the cutaneous surface indicated too little motion, and very likely retention of the results of tissue metamorphosis. A saline draught, to promote motion, was prescribed; and in the evening the child was better, with temperature 99½, and pulse fuller and slower. A warm bath was prescribed before going to bed. Next morning, temperature 98½. The indication was to increase motion in the interest of nutrition, or constructive metamorphosis, for which purpose iron, quinine and sulphuric acid were prescribed. To have honest food, and little or no trash. This evening, January 1, 1870, convalescent; exultingly tells me she has eaten a pound of beef-steak, to-day, but thinks it very funny she can't have candy, mince-pie, cake and hickory-nuts on New Year's day.

It seems to me, therefore, that these two simple physical elements of form and motion are the true foundations and corner-stones of a science of life, from a purely physical basis; and inasmuch as they absolutely cover the whole of the phenomena, or "qualities" of organic life, must, sooner or later, be so accepted by the scientific world, and sciences of physiology, pathology and therapeutics constructed in accordance; for the simple reason that they will be true, and correctly represent the facts of organic life to the mind of each student.

